# Supplementary material for: Introducing Molecular Sieve into Activated Carbon to Achieve High-Effective Adsorption for Ethylene Oxide
Source: Nanomaterials (Basel). 2024 Sep 12;14(18):1482. doi: 10.3390/nano14181482 (PMC11435392; doi:10.3390/nano14181482)
Supplement: Supplementary file 1 [file nanomaterials-14-01482-s001.zip › nanomaterials-3155689-supplementary.pdf]

Supplementary Materials

Introducing Molecular Sieve into Activated Carbon to Achieve High-Effective Adsorption for Ethylene Oxide

Feng Liu [1,2], Lingyan Qin [2,3], Pingwei Ye [2,*], Bo Yang [2], Qiong Wu [2], Li Li [2], Yuwei Dai [1], Chuan Zhou [2,*] and Sumin Li [1,*]

[1] School of Materials Science & Engineering, Jiangsu University, Zhenjiang 212013, China; liufeng001013@163.com (F.L.); dywei510@163.com (Y.D.)
[2] State Key Laboratory of NBC Protection for Civilian, Beijing 102205, China; qinlyjy@163.com (L.Q.); dahema2007goodluck@163.com (B.Y.); wuqiong1710@aliyun.com (Q.W.); lily97@buaa.edu.cn (L.L.)
[3] College of Chemical Engineering, Beijing University of Chemical Technology, Beijing 100029, China
* Correspondence: yepw2001@163.com (P.Y.); zhouc_fh@163.com (C.Z.); li_sm@ujs.edu.cn (S.L.)

Abbreviation

EtO Ethylene Oxide
AC Actived Carbon
MS Molecular Sieve
CMC Carboxymethyl Cellulose
DFT Density-Functional Theory
BJH Barrett-Joyner-Halenda
RH Relavive Humidity
SEM Scanning Electron Microscope
XRD X-ray Diffraction
TGA Thermogravimetric Analysis
FTIR Fourier Transform Infrared Spectrometer
TPD Temperature Programmed Desorption

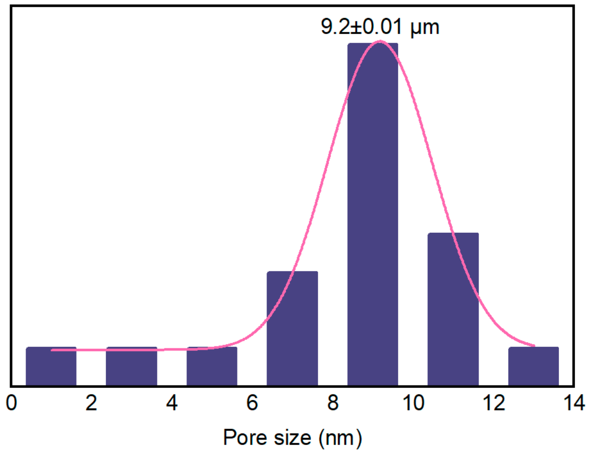

Figure S1. AC-R Pore Structure Distribution Diagram.

EtO adsorption breakthrough experiments

The breakthrough experiments are conducted on a fixed bed with a diameter of 10 mm and a

length of 20 mm. Prior to the experiments, the samples are dehydrated in a 120 °C blast furnace for 4 h. The cycling experiments follows the same procedure. EtO breakthrough experiments are conducted in a constant airflow, challenging a concentration of 500 ppm (diluted with air). The total flow rate at the inlet is kept at 500 ml min^{-1}, and the temperature and humidity of the airflow are controlled by a temperature and humidity regulator. GC8890 gas chromatograph is used to measure the inlet and outlet concentrations of EtO. When the concentration of bed exhaust gas reaches 5 ppm, it is considered that the adsorption bed has penetrated. Process and fit the experimental results, and present them in the form of breakthrough curves and fitting curves.

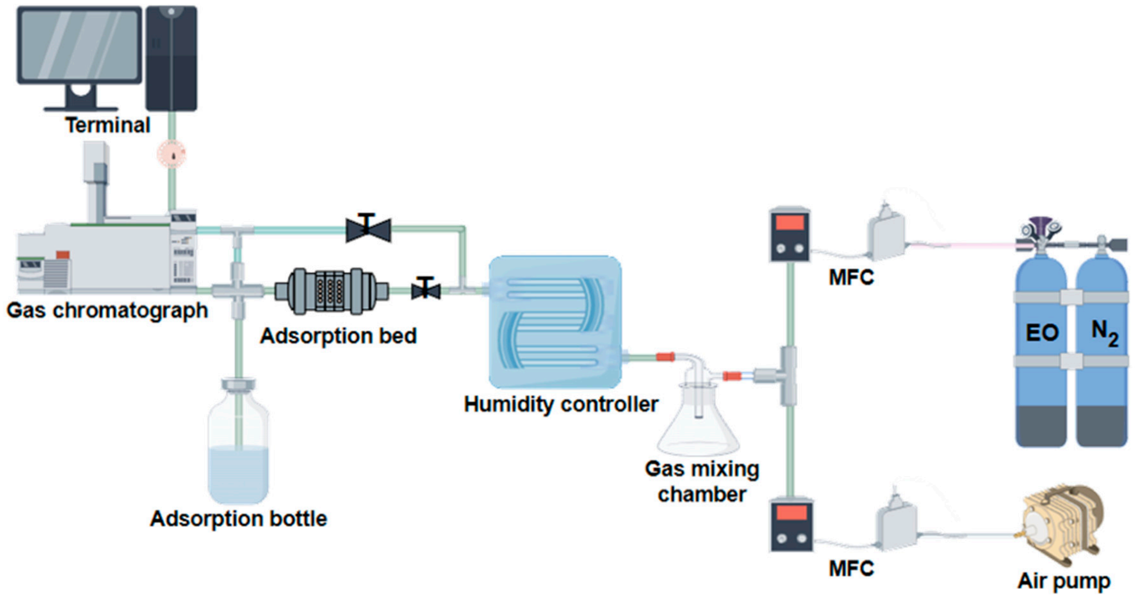

Figure S2. The Schematic illustration of dynamic adsorption device.

Adsorption performance test

In order to eliminate the influence of the volume of fixed-bed on adsorption capacity, a breakthrough experiment without adsorbent filling was carried out and the pseudo adsorption capacity was calculated [1,2]. Then, the actual adsorption capacity of the sample was derived from the difference between the total capacity and the pseudo capacity [3].

The time for EtO concentration at the outlet of the adsorption column to reach 1% of that at the inlet is defined as the breakthrough time. The breakthrough curve of EtO adsorption can be obtained and the adsorption capacity is calculated according to the following equation Eq S1:

$$q_t = \frac{V}{M}\int_{t_1}^{t_2}(C_t - C_0)\, d_t \qquad (1)$$

where q_t (mg/g) is the adsorption capacity of EtO at time t (min), V (ml/min) is the total gas flow rate, C_0 (mg/m^3) is the inlet concentration of EtO, C_t (mg/m^3) is the outlet concentration of EtO at time t and M (g) is the dosage of the adsorbent.

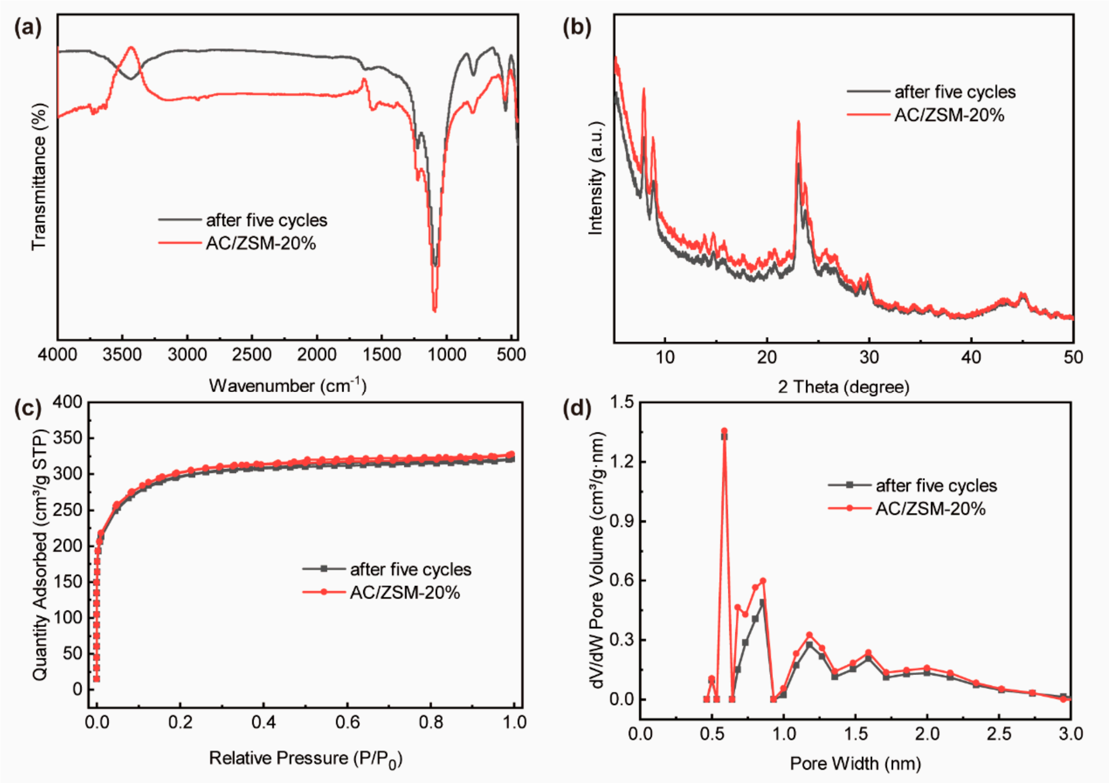

Figure S3. The FT-IR, XRD, BET and DFT pore structure distributions after five cycles.

Table S1. Specific surface area (S_{BET}), micropore surface area (S_{mic}), total pore volume (V_{tot}), micropore volume (V_{mic}) of AC-R, ZSM-R, series of AC/ZSM composites, and comparative adsorbent samples.

Sample	S_{BET} m^2/g	S_{mic} m^2/g	V_{total} cm^3/g	V_{mic} cm^3/g	Company	Country
3Å Strip molecular sieve	29.4	7.6	0.061	0.003	Shanghai Boer Chemical Reagent Co., LTD	China
4Å Strip molecular sieve	198.0	126.8	0.081	0.042	Shanghai Boer Chemical Reagent Co., LTD	China
5Å Strip molecular sieve	252.3	163.6	0.115	0.067	Shanghai Boer Chemical Reagent Co., LTD	China
3Å Spherical molecular sieve	26.4	7.6	0.014	0.003	Shanghai Maclin Biochemical Technology Co., LTD	China
4Å Spherical molecular sieve	127.0	111.8	0.098	0.043	Shanghai Maclin Biochemical Technology Co., LTD	China
5Å Spherical molecular sieve	605.7	545.4	0.241	0.209	Shanghai Maclin Biochemical Technology Co., LTD	China
13X Spherical molecular sieve	362.7	317.2	0.298	0.155	Shanghai Maclin Biochemical Technology Co., LTD	China
DX-20 coal charcoal	783.4	390.6	0.321	0.164	Shanghai Maclin Biochemical Technology Co., LTD	China
DX-09 coal charcoal	1192.7	581.9	0.498	0.247	Shanghai Maclin Biochemical Technology Co., LTD	China

Table S2. Comparison of adsorption capacity of different adsorbents for EtO at 25 °C and 0% RH.

Sample	Saturated adsorption time (min/g)	Saturated adsorption capacity (mg/g)	Source
3Å Strip molecular sieve	16.5	9.4	Commercial sample
4Å Strip molecular sieve	100	82.5	
5Å Strip molecular sieve	105.5	85.5	
3Å Spherical molecular sieve	30.5	15.5	
4Å Spherical molecular sieve	99	82.4	
5Å Spherical molecular sieve	101.5	83.9	
13X Spherical molecular sieve	104.5	85.4	
MZ-20 carbon	49.5	39.0	
DX-09 coal charcoal	62.5	48.4	
AC-R	77.5	56.1	this work
ZSM-R	110.5	97.8	
AC/ZSM-10%	142.5	118.2	
AC/ZSM-20%	180.5	145.1	
AC/ZSM-30%	160.5	124.4	

Table S3. Kinetic models used in this study.

Pseudo-first-order	$q_t = q_e[1 - e^{(-k_1 t)}]$	[1]
Pseudo-second-order	$q_t = \frac{k_2 q_e^2 t}{[1 + q_e k_2 t]}$	[2]
Boltzmann	$q_t = \frac{A_1 - A_2}{1 + e^{(q_e - q_0)/d_q}} + A_2$	[3]

Table S4. Parameters of the kinetic equation for the adsorption of EtO by various quality proportion of AC/ZSM at 50% humidity.

Kinetics equation	Parameters	AC-R	AC/ZSM-10%	AC/ZSM-20%	AC/ZSM-30%	ZSM-R
Pseudo-first-order	q_e (mg/g)	47.14	56.04	113.13	100.13	101.61
	k_1 (min^{-1})	0.026	0.023	0.010	0.011	0.012
	Adj.R^2	0.985	0.986	0.993	0.990	0.987
Pseudo-second-order	q_e (mg/g)	18.34	22.88	35.95	32.88	35.41
	k_2 (g·(mg·/min))	-4.42×10^{44}	-2.11×10^{43}	-1.01×10^{45}	-5.33×10^{44}	-7.95×10^{44}
	Adj.R^2	0.047	0.032	0.017	0.024	0.020
Boltzmann	q_e (mg/g)	15.46	16.20	27.60	25.38	25.39
	A_1	-5.05	-9.23	-21.02	-14.69	-23.87
	A_2	34.13	41.67	75.83	63.86	74.47
	Adj.R^2	0.997	0.998	0.998	0.998	0.999

Table S5. Parameters of the kinetic equation for the adsorption of EtO by AC/ZSM-20%.

Kinetics equation	Parameters	0%RH	15%RH	50%RH	75%RH	95%RH
Pseudo-first-order	q_e (mg/g)	272.74	173.02	101.61	72.133	53.191
	k_1 (min^{-1})	0.004	0.006	0.012	0.017	0.023
	Adj.R^2	0.991	0.990	0.987	0.986	0.987
Pseudo-second-order	q_e (mg/g)	64.59	46.22	35.41	28.67	21.59
	k_2 (g/(mg · min))	3.19×10^{-44}	-3.71×10^{43}	-1.01×10^{45}	-2.38×10^{44}	3.84×10^{45}
	Adj.R^2	0.009	0.012	0.017	0.024	0.035
Boltzmann	q_e (mg/g)	57.52	40.17	27.60	20.98	15.09
	A_1	-44.62	-30.46	-21.02	-13.91	-9.28
	A_2	159.63	110.72	75.83	55.31	39.08
	Adj.R^2	0.998	0.998	0.998	0.998	0.998

References

[1] C. Zhou, K. Zhou, H. Li, X. Xu, B. Liu, H. Li, Z. Zeng, W. Ma, L. Li. Pressure swing adsorption properties of activated carbon for methanol, acetone and toluene. Chem. Eng. J. **2021**, 413, 127384-127396.

[2] S. H. Hong, M.Y. Jung, C. H. Lee. Performance and dynamic behavior of H_2 layered-bed PSA processes using various activated carbons and zeolite LiX for steam methane reforming gas. Chem. Eng. J. **2023**, 473, 144942-144960.

[3] Z. Guo, J. Huang, Z. Xue, X. Wang. Electrospun graphene oxide/carbon composite nanofibers with well-developed mesoporous structure and their adsorption performance for benzene and butanone. Chem. Eng. J. **2016**, 306, 99-106.
